# The Burden of Hepatocellular Carcinoma in Non-Alcoholic Fatty Liver Disease: Screening Issue and Future Perspectives

**DOI:** 10.3390/ijms20225613

**Published:** 2019-11-09

**Authors:** Grazia Pennisi, Ciro Celsa, Antonina Giammanco, Federica Spatola, Salvatore Petta

**Affiliations:** 1 Sezione di Gastroenterologia e Epatologia, PROMISE, University of Palermo, 90127 Palermo, Italy; celsaciro@gmail.com (C.C.); federicaspatola1991@gmail.com (F.S.); 2 Sezione di Astanteria e MCAU, PROMISE, University of Palermo, 90127 Palermo, Italy; agiamman@gmail.com

**Keywords:** non-alcoholic fatty liver disease, NAFLD, hepatocellular carcinoma, HCC, PNPLA3, TM6SF2, miRNA, micro RNA, lncRNA, long non-conding RNA

## Abstract

In recent decades, non-alcoholic fatty liver disease (NAFLD) has become the most common liver disease in the Western world, and the occurrence of its complications, such as hepatocellular carcinoma (HCC), has rapidly increased. Obesity and diabetes are considered not only the main triggers for the development of the disease, but also two independent risk factors for HCC. Single nucleotide polymorphisms (such as PNPLA3, TM6SF2 and MBOAT7) are related to the susceptibility to the development of HCC and its progression. Therefore, an appropriate follow-up of these patients is needed for the early diagnosis and treatment of HCC. To date, international guidelines recommend the use of ultrasonography with or without alpha-fetoprotein (AFP) in patients with advanced fibrosis. Furthermore, the use of non-invasive tools could represent a strategy to implement surveillance performance. In this review, we analyzed the main risk factors of NAFLD-related HCC, the validated screening methods and the future perspectives.

## 1. Introduction

Non-alcoholic fatty liver disease (NAFLD) is a metabolic disorder including a spectrum of pathological conditions ranging from simple steatosis (NAFL) to steatohepatitis (NASH) and cirrhosis.

NAFLD represents the most common liver disease worldwide, with a global prevalence of 25% [1]. Environmental (especially diabetes and obesity) and genetic factors seem to influence geographical distribution of NAFLD, which is higher in Asia and South America and lower in Africa.

The pandemic of NAFLD represents the heaviest burden among modern liver diseases, not only due to its epidemiology, but also because of the risk of progression to cirrhosis and its complications, such as hepatic decompensation, hepatocellular carcinoma (HCC) and death. Ekstedt et al. [2] showed an increased overall mortality (HR 1.29), with increased risk of cardiovascular disease (HR 1.55) and HCC (HR 6.55) in biopsy-proven NAFLD patients compared to a general population.

## 2. The Burden of NAFLD-Related HCC

HCC represents the fifth most common cancer worldwide. To date, it is the second most frequent cause of cancer-related death globally, with a progressive growing trend. [3]

In more than 80% of cases, HCC develops in cirrhosis with different underlying etiologies, mainly viral hepatitis (HCV–HBV) and fatty liver (alcoholic and non-alcoholic related). While the epidemiological data concerning HCC in viral and alcoholic hepatitis are consolidated, there is a lack of strong epidemiological data concerning the incidence and prevalence of HCC in NAFLD.

In recent last years, we observed a different pattern of incidence of HCC among the different etiologies of liver disease. Several international studies [4,5] conducted in the last decade confirmed this evidence. Goldberg and colleagues collected data of 1853 patients with cirrhosis and HCC, showing a decrease in the percentage of HCV- and alcohol-related HCC and an increase in NAFLD-related HCC [6]. Liu et al. recently showed a global increase of incident cases of liver cancer of 114.0%, from 471,000 in 1990 to 1,007,800 in 2016. Interesting highlights are the geographical changes in epidemiology: Decrease in incidence was observed in some regions due to the control of HBV and HCV infections, but an increase was observed in countries with a high socio-demographic index, including the Netherlands, the UK, and the USA, with this issue probably being related to NAFLD. [7].

These epidemiological and etiological changes also reflect a reverse trend in patients on the waitlist for liver transplant (LT). Younossi et al. evaluated 158,347 LT candidates and 26,121 (16.5%) of this had HCC. The proportion of HCC increased from 6.4% (2002) to 23.0% (2016) (*p* < 0.0001). The study showed not only a growing incidence of HCC during follow-up, but also a changing trend of etiology. Over the study period, HCV remained the most common etiology for HCC (65%). The proportions of HBV and alcohol-related HCC remained stable (both trend *p* > 0.10), HCV-related HCC decreased 3.1-fold (*p* < 0.0001), while the NASH one in HCC increased 7.7-fold (from 2.1% to 16.2%; *p* < 0.0001). [8] Similar results were observed in the European Liver Transplant Registry (ELTR) during Direct-Acting Antivirals agents (DAAs) era, on 60,527 LT candidates (28.3% with HCC) between January 2007 to June 2017 [9].

US data showed an increase in age-standardized NAFLD-related mortality compared to others etiologies during the last 10 years (from 2007 to 2016). [10]. This evidence can probably be found not only for steady increase of NAFLD incidence, but also for the growing incidence of HCC due to NAFLD. In this way, Estes et al. developed a modeling approach to forecast the current and future burden of disease due to NAFLD in the United States. NAFLD-related HCC prevalence is estimated to increase, ranging from 47% in Japan to 130% in the US; similarly, its incidence is also estimated to increase, ranging from of 44% in Japan to 122% in the US [11].

Overall, these data suggest that NAFLD is going to be the most relevant etiology of HCC in the coming years. The explanation of the epidemiological changes discussed above may be related to the development of antiviral therapies (i.e., DAAs for HCV infection) that were shown to be effective in decreasing the risk of HCC occurrence and hepatic decompensation and to the rapid worldwide increase of the prevalence and the incidence of metabolic disorders, such as diabetes and obesity. In the future, epidemiological data able to reliably quantify the alarming growth of NAFLD-related HCC will be needed, especially in patients without cirrhosis, as well as properly designed studies for assessing the impact of therapy, both lifestyle and pharmacological approaches, on the risk for the development of HCC.

## 3. The Impact of Metabolic and Genetic Risk Factors

**Figure 1:**
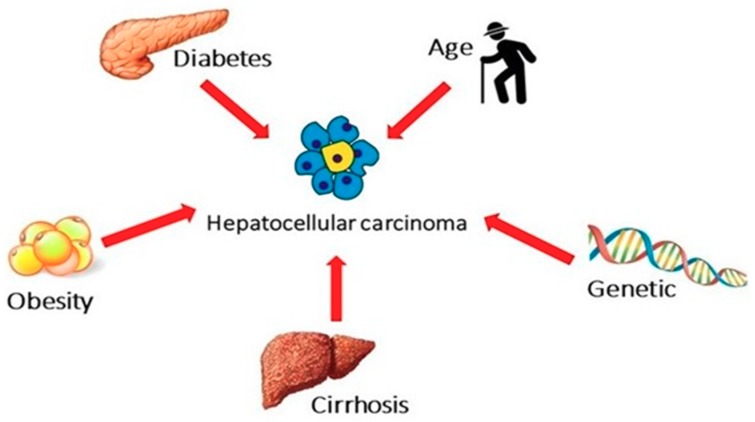
Metabolic and genetic risk factors to development NAFLD-related HCC.

The strong association between NAFLD and metabolic and genetic risk factors is widely known, and these present themselves risk factors for cancer (not only HCC) (Figure 1).

### 3.1. Obesity

Large cohort studies showed a strong association between obesity and cancer incidence (both overall and for specific sites) [12].

Although the pathogenic molecular mechanisms are not completely clear, chronic low-level inflammation associated with obesity plays a key role in damaging DNA and reducing repair [13]. Furthermore, obesity is associated with increased cancer-related mortality [14].

Particularly, in a large prospective cohort study assessed the relationship between BMI and the risk of death from cancer in 900,053 subjects followed-up for 16 years [15], the authors showed a significant positive linear trend in death rate with increasing BMI for different cancer sites, including HCC.

Interestingly, a different magnitude of risk according to sex was observed, and differences were more pronounced for higher BMI values: The relative risks for death from HCC in patients with severe obesity (BMI ≥ 35 kg/m^2^) were 4.52 in men and 1.68 in women, compared with normal-weight subjects. The gender difference in the relationship between BMI and HCC was further assessed in a meta-analysis of 17 studies, including 18,225 patients [16]. The positive association between overweight and obesity with HCC was confirmed (RRs 1.16 (1.08–1.25) and 1.83 (1.60–2.09) respectively), using normal weight as comparison, and subgroup analyses of obese patients showed a RR for liver cancer of 2.04 (1.70–2.44) in men and 1.56 (1.37–1.78) in women, with a significant *p*-value for interaction (*p* = 0.02). Conversely, interaction analysis showed no significant differences (*p* for interaction = 0.47) between overweight men and women (RRs 1.18 (1.01–1.30) and 1.11 (1.00–1.24), respectively).

A sensitivity analysis conducted on 8 studies showed a nonlinear dose-response relationship between BMI and risk of HCC, that increased of 4% for each 1 kg/m^2^ increase in BMI. An explanation for this gender difference could be related to the possible effects of estrogens in protecting females from developing HCC as the administration of estrogens before the initiating event resulted protective against HCC in experimental liver carcinogenesis models [17].

### 3.2. Diabetes

Diabetes represents another emerging risk factor for both NAFLD/NASH and for HCC.

A large prospective cohort study conducted in 173,643 patients with diabetes and 650,620 without diabetes revealed an incidence rate for HCC that was significantly higher among patients with diabetes (2.39 versus 0.87 per 10,000 person-years. *p* < 0.0001) [18].

Multivariate analysis showed that diabetes was associated with a greater than 2-fold increase in risk of HCC, after adjustment for anthropometric and demographic factors, and comorbidities. A sensitivity analysis after exclusion of patients with HCV, HBV, alcohol consumption and fatty liver confirmed the independent association between diabetes and HCC. Although it should be considered that these data have been obtained in patients identified at hospitals of Veterans Affairs, similar results were observed in a US population-based study using Surveillance, Epidemiology and End-Results (SEER) registries [19]. Specifically, after adjustment for well-known HCC risk factors, diabetes was associated with a threefold increase in the risk of HCC.

The magnitude of this association decreased, but still remained significant, in the subgroup of patients without major risk factors. Similar results were observed in a Japanese retrospective population cohort study on 6,508 patients with ultrasound (US)-diagnosed NAFLD followed-up for a median period of 5.6 years [20]. Although the incidence of HCC was quite low, the cumulative rate of HCC was significantly higher in the group of patients with diabetes in comparison with those without diabetes, and it was respectively 3.42% versus 0.10% at 12 years. These initial findings have been subsequently confirmed by a systematic review with meta-analysis including 25 cohort studies that showed an increased incidence of HCC in diabetic patients in comparison with those without diabetes, with a pooled RR of 2.01 (95% CI 1.61–2.51) [21].

Although heterogeneity among studies was high and significant, after stratification for geographic area, HBV or HCV infection, alcohol use, cirrhosis and duration of follow-up, the association between diabetes and increased incidence of HCC remained significant. Notably, authors also assessed the HCC mortality, that resulted increased in diabetic patients, compared with those without diabetes, with a pooled RR of 1.56 (95% CI 1.30–1.87). An Italian multicenter prospective study compared the clinical features between HCC patients with NAFLD (*n* = 145) and HCV-related liver disease (*n* = 611) [22].

Not surprisingly, the features of metabolic syndrome (including diabetes) were significantly more prevalent among HCC patients with NAFLD, as well as other risk factors for HCC such as age, male sex, and alcohol consumption. Interestingly, only about 50% of HCC patients with NAFLD had cirrhosis, and the absence of HCC surveillance in these patients could explain the larger HCC sizes and the more frequent infiltrative pattern observed in NAFLD patients, compared to HCV patients. Although unadjusted analysis showed a significantly shorter survival in HCC patients with NAFLD than in those with HCV, after propensity score matching no significant differences were observed in survival between the two groups. More recently, the association between diabetes and HCC was assessed in 354 Mayo Clinic patients with NASH cirrhosis [23].

Multivariate analysis showed that diabetes (HR 4.2 95% CI 1.2–14.2, *p* = 0.02), age and low albumin significantly predicted the development of HCC, differently from other metabolic risk factors, such as BMI. Overall, the data presented provide a strong evidence that obesity and diabetes increase the risk for HCC regardless of the presence of liver disease and they could influence the mortality of these patients limiting the access to curative treatments for HCC, but the impact of these risk factors among NAFLD patients should be further assessed in well-designed prospective studies and pathogenic mechanisms should be understood more clearly, aiming to improve the cost-effectiveness of surveillance programs and to develop novel targeted therapies.

### 3.3. Genetics Findings

Epidemiological and genetic studies indicate a strong pattern of heritability that may explain some of the variability in NAFLD phenotype and risk of progression. Among the other risk factors, inherited factors contribute to HCC susceptibility, and strong familial aggregation is observed [24]. To date, at least three common genetic variants in the patatin-like phospholipase domain-containing protein 3 (PNPLA3), transmembrane 6 superfamily member 2 (TM6SF2), and membrane-bound Oacyltransferase domain-containing 7 (MBOAT7) genes have been robustly linked to NAFLD in the population. The function of these genes revealed novel pathways implicated in both the development and progression of NAFLD. Table 1 summarizes some of the most relevant findings about the role of genetic in NAFLD-related HCC.

#### 3.3.1. PNPLA3

The rs738409 C > G variant in the PNPLA3 gene, encoding an I148M mutation, is an independent genetic risk factor for NAFLD, associated with the wide spectrum of severity of NAFLD [25]. In the initial studies, the PNPLA3 I148M variant was associated with intrahepatic fat content and subsequently found to be associated with NASH, hepatic fibrosis, and hepatocellular carcinoma (HCC) [26,27,28]. In a cross-sectional study that evaluated the role of PNPLA3 rs738409 [G] risk allele, genotype frequencies were compared between 100 European Caucasian patients with NAFLD-related HCC and 275 controls with biopsy proven NAFLD without HCC, showing that CG and GG genotype was associated with a 2- and 5-fold increased risk for HCC, respectively, in comparison with the CC genotype, by multivariate analysis [29]. More recently, we performed a prospective study on 471 patients with histologically diagnosed NAFLD demonstrating an independent association between PNPLA3 C > G variant and HCC, by multivariate analysis (HR 2.10, 95% CI 1.03-4.29; *p* = 0.04) [30]. The PNPLA3 I148M variant is also associated with increased risk of fibrosis progression and HCC in alcoholic liver disease and hepatitis C-related cirrhosis and independent of steatosis [27,28], suggesting a potential direct contribution of the variant to fibrogenesis and carcinogenesis that are unrelated to intrahepatic triglyceride accumulation [31]. These results suggest that PNPLA3 genotype could be used as a tool to stratify the risk for HCC in combination with other well-known risk factors, e.g., liver function tests, portal hypertension, and its surrogates. However, an external validation of these results is needed before the PNPLA3 genotype could be used in clinical practice.

#### 3.3.2. TM6SF2

Similar to the PNPLA3 I148M variant, the rs58542926 C > T variant in the TM6SF2 gene, encoding an E167K mutation, was shown to be associated with hepatic steatosis and also with increased risk of progressive liver disease and fibrosis [32,33,34,35], but its direct role in HCC predisposition is disputed [35,36].

#### 3.3.3. MBOAT7

The rs641738 C > T variant of the MBOAT7 gene was identified in alcoholic-related cirrhosis and subsequently confirmed to increase risk of hepatic steatosis, progressive liver disease in NAFLD [37,38], and HCC risk in non-cirrhotics with NAFLD, as well as non-cirrhotic chronic hepatitis C and alcoholic liver disease [39]. An Italian study assessed the role of this variant, showing an association between the carriage of T allele and HCC (OR 1.65, 95% CI 1.08–2.55), especially in patients without advanced fibrosis, suggesting that MBOAT7 variation predisposes to HCC development, particularly in non-cirrhotic patients [39]. Interestingly, a number of PNPLA3, TM6F2 and MBOAT7 risk variants significantly predicted HCC, independently of other clinical factors, although this model did not significantly improve the accuracy in the prediction of the risk for HCC. However, it must be emphasized that these data were provided by cross-sectional studies, and that the identification of a single genetic mutation critically involved in the increased susceptibility to develop HCC is difficult, considering that inheritance of NAFLD is polygenic. Furthermore, prospective data evaluating the impact of selected single nucleotide polymorphisms (SNPs) on HCC occurrence are still lacking.

All these data suggest that genetic variants predisposing to hepatic fat accumulation promote hepatic carcinogenesis. Indeed, hepatocellular fat accumulation represents a key feature of hepatic carcinogenesis [40,41].

#### 3.3.4. Alpha-fetoprotein

AFP is a 70-kD glycoprotein synthetized by fetal yolk sac and fetal liver. Its levels markedly decrease during the first year of life; therefore, tumors arising from endodermal lining of hepatic diverticulum can be associated with an increase in serum levels. However, it should be considered that inflammatory activity in chronic hepatitis can be associated with fluctuations of serum levels [42]. In patients with HCC, AFP levels range from normal to above 10,000 ng/mL, but about one third of patients could have low AFP levels, also in the presence of advanced HCC [43]. Notably, a persistent elevation in AFP levels represents a risk factor for the development of HCC, allowing the identification of selected high-risk populations [44]. It should be considered that the accuracy of AFP in surveillance was suboptimal, making a translation of its diagnostic role in the surveillance setting misleading [45]. Values of 20 ng/mL showed good sensitivity and low specificity, while, as expected, levels above 200 ng/mL were associated with high specificity but lower sensitivity [46], although it must be observed that these data were obtained in patients with viral hepatitis and data about the role of AFP in surveillance and diagnosis of NAFD-related HCC are lacking. The role of the association between ultrasound and AFP will be discussed below. On the other hand, AFP is a useful tool for the prediction of the prognosis of patients with HCC [47]. The use of AFP isoforms has been proposed to improve the accuracy of AFP in the early detection of HCC. According to the changes in the glycan terminal chain, eleven isoforms of AFP have been identified. Specifically, it has been shown that a specific isoform for HCC is able to bind lectins lens culinaris agglutinin-A (AFP-L3). A Japanese study showed that AFP-L3 is positive in about one third of HCC smaller than 2 cm and that its appearance in serum could anticipate the radiological detection of the tumor [48]. The role of AFP-L3 was subsequently assessed in a North American study, which demonstrated a high specificity of AFP-L3 in patients with elevated AFP levels [49]. The fractionating of AFP in four band using isoelectric focusing represents another promising tool to improve the accuracy of this biomarker, although technical complexity and costs limit its spread in the clinical daily practice [50].

## 4. The Issue of Screening

The identification of NAFLD patients needing a systematic screening for HCC still represents a critical issue. This is related to the poor knowledge of the molecular pathways involved in hepatocarcinogenesis, the wide heterogeneity existing among NAFLD patients, and the coexistence of other risk factors for HCC, in addition to chronic liver disease. For this reasons, predictive biomarker models able to identify specific high-risk subgroups remain an unmet clinical need, considering the increasing incidence of NAFLD in general population and the possibility of the development of HCC also in the absence of advanced fibrosis or cirrhosis. Therefore, the identification of personalized risk profiles of HCC in NAFLD patients represents a clinical and methodological challenge. 

### 4.1. Who Screen

The correct identification of NAFLD patients to be screened requires the accurate estimation of the incidence of HCC in patients with or without cirrhosis.

Furthermore, among NAFLD noncirrhotic patients, incidence of HCC could be extremely heterogeneous according to the technique (US versus histology) employed to obtain the diagnosis of NAFLD. Although it has been reported in retrospective studies that HCC can also occur in NASH without cirrhosis [51,52], large-scale prospective studies are lacking. This could lead to an underestimation of the risk for HCC in NASH without cirrhosis and subsequently in the exclusion of higher risk patients from surveillance programs. A recent meta-analysis of 7 studies including 1191 patients with noncirrhotic NASH and 21,868 controls with other etiologies of chronic liver disease without cirrhosis has shown a prevalence of HCC in noncirrhotic NASH of 38% versus 14.2% in control group (*p* < 0.001), with an increased risk for HCC of 26% [53].

However, the significant heterogeneity observed among the included studies and the lack of data on fibrosis stages in noncirrhotic patients limit the usefulness of the results of this meta-analysis. Among noncirrhotic patients, the way in which the diagnosis of NAFLD is performed could influence the incidence of HCC, potentially reflecting a selection bias whereby patients who underwent liver biopsy were those with more severe liver disease, and therefore with higher risk for HCC. In a Japanese population study conducted on more than 6,000 patients with US-proven NAFLD, only 16 subjects developed HCC over a 5.6-year follow-up, with a crude rate of 0.25% and a yearly cumulative HCC incidence of 0.043%. Interestingly, the incidence of HCC was significantly higher in patients with higher AST to Platelet ratio index (APRI) than in those with low APRI (*p* < 0.001), suggesting a potential usefulness of this non-invasive tool in this clinical setting. However, the lack of a control group is a limitation of this study, as well as the transferability of this data to Western populations. In this sense, a retrospective multicenter cohort study conducted in 130 hospitals of Veterans Health Administration in the USA compared 296,707 US-proven NAFLD patients with 296,707 age- and sex-matched controls [54].

Incidence of HCC in NAFLD was 0.21/1000 person years and HCC was significantly more frequent among NAFLD patients in comparison with controls, also after adjustment for race and metabolic syndrome (HR 7.62. 95% CI 5.76–10.09). Notably, the incidence of HCC in NAFLD patients without cirrhosis (0.08/1000 person years) was higher than that observed in the control group without NAFLD (0.02/1000 person years), and the patients with higher risk were male, older, Hispanics and cirrhotics.

However, in patients without cirrhosis, the overall risk was too low to justify HCC screening. Along these lines, our data on 471 biopsy-proven NAFLD patients followed-up for a median period of 64.6 months showed that 13 patients (2.7%) developed HCC and all of them had F3–F4 fibrosis, was independently associated with HCC occurrence, together with the PNPLA3 G > C variant in the multivariate analysis [30].

The absence of HCC among patients with fibrosis F0–F2 in our cohort could be explained by the young mean age of the included patients and by the moderately long follow-up.

Finally, the risk for HCC in patients with NASH cirrhosis was specifically investigated and compared with those of HCV-related cirrhosis in a prospective study over a median follow-up of 3.2 years. HCC occurred in 25/195 of patients with NASH cirrhosis and in 64/315 of those with HCV-related cirrhosis. Yearly cumulative incidence was not significantly higher in HCV-related cirrhosis than in NASH cirrhosis (4% vs. 2.6%, respectively, *p* = 0.09) and multivariate analysis identified older age and any alcohol consumption as independent risk factors for the occurrence of HCC [55].

According to previously reported data, patients with NAFLD but without cirrhosis seem to have a risk of HCC that is higher compared to subjects without liver disease and those with other etiologies of noncirrhotic chronic liver disease, but considering that the surveillance for HCC is cost-effective if incidence is higher than 1.5% per year, the risk for HCC in patients with noncirrhotic NAFLD appears to be too low to justify an extensive surveillance program over the entire NAFLD population. If there is no doubt that surveillance should be performed in patients with compensated cirrhosis, it is not clear whether noncirrhotic F3 patients should also be included and European guidelines express a weak recommendation on this issue [56].

At the same time, not all cirrhotic patients have the same risk for HCC, and six-month abdominal US do not represent a “one size fits all” approach. The development of scoring systems based on the combination of demographic, anthropometric, clinical and biochemical features could be useful to stratify the risk for HCC. Since the incidence of HCC is significantly different according to the etiology of liver disease, the Toronto HCC index was developed to predict the disease-specific risk for HCC in patients with cirrhosis [57].

Points are assigned according to age, etiology, gender and platelet count, allowing stratification into low-, medium- and high-risk subgroups. Particularly, authors observed in the derivation cohort a 10-year cumulative HCC incidence of 3%, 10% and 32%, respectively, and they validated these data in an external cohort.

More recently, Ioannou et al. developed a simple model to estimate HCC risk using data from 7068 patients with NAFLD cirrhosis (1,278 with incident HCC) within the Veterans Affairs healthcare system [58]. The model includes simply available covariates, such as age, gender, diabetes, BMI, platelets, albumin and AST to ALT ratio, exhibiting an area under the receiver operating characteristic curve (AUROC) of 0.75. The main limitation of such a model is the poor accuracy in the prediction of the risk at single patient’s level, as suggested by the relatively low value of AUROC (which was similar for patients with alcohol-related cirrhosis). However, authors showed that a risk-based screening according this prediction model was associated with a higher standardized net benefit in comparison with an approach to screen all.

Finally, an approach to HCC risk profiling based on the combination of clinical (i.e., diabetes, obesity, severity of portal hypertension), biochemical (APRI), histological (severity of fibrosis) and genetic (i.e., PNPLA3 and MBOAT7) features could identify personalized risk profiles associated with the development of HCC in patients with NAFLD and further data will be needed to identify NAFLD patients without cirrhosis at higher risk of HCC for whom a surveillance program could result cost-effective.

### 4.2. How to Screen

**Figure 2:**
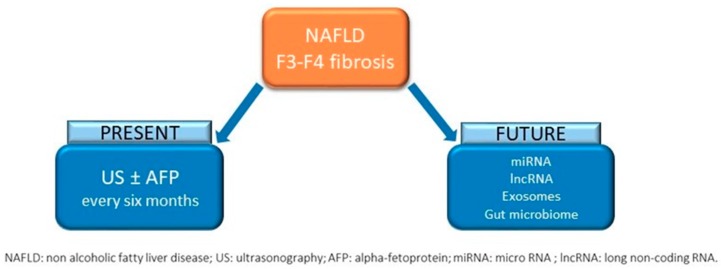
HCC surveillance in patients with NAFLD and advanced fibrosis: Present and future perspectives.

According to the data mentioned above, it is still not clear which NAFLD patients without cirrhosis should undergo HCC surveillance and among those with cirrhosis which subgroups are at higher risk for HCC, needing a more stringent surveillance program (Figure 2).

At the same time, it is not even clear what technique should be used to screen NAFLD patients for HCC. If several national and international guidelines agree on the use of 6-months abdominal US, different are the recommendations about the use of serum biomarkers (i.e., alpha-fetoprotein [AFP]).

While European guidelines [56] state that cost-effectiveness of available biomarkers is too low to recommend a routine use in surveillance, American guidelines recommend the use of US with or without AFP and Korean guidelines support the use of 6-month assessment of AFP levels, in combination with US [59,60].

The performance of US, with or without AFP, in detecting HCC, was assessed in a meta-analysis of 32 studies, including 13,367 patients, that reported sensitivity and specificity of different surveillance strategies [61]. Pooled sensitivity of US for any HCC stage resulted quite good (84%, 95% CI 76%–92%), but it decreased to a modest value with a wider confidence interval (47%, 95% CI 33%–61%), restricting the analysis to the detection of only early stage HCC. Eighteen out of 32 studies compared the sensitivity of US with or without AFP for the detection of HCC at any stage: the addition of AFP to US resulted in an improvement of the sensitivity (97%, 95% CI 91%–99%) in comparison with US alone (78%, 95% CI 67%–86%).

However, the increased sensitivity associated with the combination of US and AFP affected the specificity, increasing the rate of false positive results. Restricting the analysis to only studies (*n* = 8) that assessed the detection of early HCC, a significant superiority of the combination of US and AFP over US alone was observed in terms of sensitivity (63% vs. 45%, respectively, *p* = 0.002), but, not surprisingly, at the cost of a decrease in terms of specificity.

The adherence to the HCC surveillance program represents another critical issue, as it has been shown that it could have an impact on survival and other relevant outcomes.

A systematic review with a meta-analysis of 22 studies (19,511 patients) showed a poor overall adherence rate (52%, 95% CI 38%–66%) with a significant heterogeneity within the included studies and compared the adherence rate among different etiologies of liver disease, showing no significant differences [62]. Study design resulted the only study-level covariate significantly associated with screening adherence by multivariate meta-regression, and prospective studies were associated with a significantly higher adherence rate compared to retrospective studies. Conversely, retrospective data from ITALICA cohort revealed not only an increase during the two last decades of “non-viral” HCC cases, but also a lower proportion of NASH-related HCC diagnosed during regular 6-month surveillance in comparison with HCV-related HCC (respectively, 39% vs. 68%) [63]

In consideration of the underuse of the regular surveillance program and its potential impact on survival, research focused on how to improve the adherence, for example through the use of a mailed outreach strategy or navigation strategies (such as active encouragement of participation to surveillance and the identification of the barriers to surveillance). A randomized controlled trial compared these two approaches with the usual care in 1800 patients with chronic liver disease and different ethnicity (16.6% had NASH as etiology and 79.6% of the overall cohort had cirrhosis) [64]. In the intention-to-treat analysis, the primary outcome (defined as the completion of abdominal imaging every 6 months for 18 months) was reached in 7.3% of usual care patients, in 17.8% of outreach-alone patients and in 23.3% of outreach/navigation patients. Particularly, a significant increase of 16% in the surveillance completion rate was observed in patients underwent the combined approach of outreach and navigation, compared to the usual approach. The ethnicity did not have a significant impact on the primary outcome, and secondary outcome (the proportion of patients with a diagnosis of early stage HCC) was not significantly different among the three intervention groups, although the study was not powered to find differences in the tumor stage at diagnosis. However, patients underwent surveillance had a significantly higher proportion of early stage HCC in comparison with those diagnosed incidentally or after the appearance of symptoms. These data suggested that the combination of mailed outreach invitations with patient navigation is an effective strategy to improve the adherence to HCC surveillance screening over a period of 18 months. Further studies will be needed to prove the effectiveness of this approach in other health systems and in cohorts of patients with a higher proportion of NASH as etiology of liver disease.

Effectiveness of US in detecting early HCC stages is another critical issue, as it was reported that US sensitivity could be very low (32%) in a clinical practice setting [65]. The inadequacy of US sensitivity lead to an increase of the proportion of patients diagnosed with late stages HCC, resulting in a dismal prognosis. The identification of the factors associated with US failure in the detection of HCC could be useful to select subgroups of patients which should be surveilled with alternative imaging techniques or to improve the ultrasound imaging acquisition. A retrospective cohort study was conducted on 941 patients with cirrhosis (11.7% with NASH as etiology) to assess the adequacy of US and to identify clinical factors associated with inadequate quality of US in surveillance [66]. The quality of US was defined according to a subjective evaluation by expert radiologists in terms of visualization of the entire liver and exclusion of focal lesions, including HCC. Interestingly, US was considered inadequate in about 20% of cases, and multivariate analysis showed that increasing BMI class and NASH were independently associated with an inadequate US quality. These findings could be explained considering that obesity and the accumulation of subcutaneous fat affect the obtainment of high-quality US images of the whole liver and that liver steatosis associated with NASH impairs the attenuation of US pulse and the visualization of possible focal lesions. These results were largely confirmed by a single-center study conducted on 352 patients referred to liver transplant with known HCC [67].

Comparing the US sensitivity to that of computed tomography (CT) or magnetic resonance imaging (MRI), authors showed that obesity and NASH were significantly associated with a lower US sensitivity. Particularly, US sensitivity was 76% in obese patients (vs. 87% in non-obese patients, *p* = 0.01) and 59% in NASH patients (vs. 84% in other etiologies, *p* = 0.02).

These data appear to be relevant as the burden of the obesity and of NASH-related HCC is rapidly increasing. Alternative imaging techniques such as CT or MRI may resolve these issues in the subgroups of patients prone to failing US surveillance, but both costs and radiation exposure should be assessed before these techniques can be extended on large scale surveillance programs. For these reasons, non-invasive accurate biomarkers urgently need to be combined with available imaging techniques and to improve the quality of HCC surveillance in NAFLD high-risk patients.

## 5. Prevention Strategies

The development of HCC in patients with NAFLD without cirrhosis suggests a NAFLD-specific mechanism of carcinogenesis that is probably independent of hepatic fibrosis [68].

### 5.1. Lifestyle Interventions

Because obesity and type 2 diabetes are independent risk factors of HCC (see above), the correction of either could be a key strategy for preventing development of HCC in patients with NAFLD. To support this hypothesis, in a meta-analysis of 13 case-control and 13 cohort studies, diabetes was associated with increased HCC risk (OR, 2.5 and HR, 2.5, respectively) [69]. Furthermore, high body mass index (BMI) was significantly associated with liver cancer risk (HR, 1.19 per BMI 5 kg/m^2^) in 5 million subjects registered in the Clinical Practice Research Datalink [70]. Therefore, lifestyle interventions could be useful in improving obesity and type 2 diabetes, and lifestyle interventions may prevent HCC. This finding is suggested by observational studies. A meta-analysis of 19 studies, involving 1,290,045 individuals, reported that healthy eating, especially a diet rich in vegetables, may reduce HCC risk (RR, 0.72) [71]. In a prospective cohort of 428,584 subjects, higher physical activity was associated with lower HCC risk (HR, 0.69) [72]. Specifically, Turati et al. showed, that adequate adherence to a Mediterranean diet was protective against HCC in two cohorts of subjects—513 patients with HCC and 722 controls (*p* < 0.001) [73]

### 5.2. Statins and Metformin

In addition to the correction of lifestyle, use of pharmacological therapy, such as statins and metformin, for the prevention of HCC appears to be interesting. Statins have a variety of pleotropic anti-neoplastic, in addition to cholesterol-lowering, effects. Statins inhibit oncogenic pathways, such as Myc [74], nuclear factor κB (NF-κB), tumor necrosis factor (TNF)-mediated IL6 production [75], extracellular signal-regulated kinase 1/2 (ERK1/2) [76], whereas adenosine monophosphate-activated protein kinase (AMPK) and p38/mitogen-activated protein kinase (MAPK) pathways are activated [77,78], and they induce p53-dependent apoptosis [79]. Statins also inhibit fibrogenic hepatic stellate cell activation via nitric oxide synthase, hepatocyte paracrine signals [80], endothelial cells [81], induction of sterol regulatory element-binding protein 1 (SREBP-1) and peroxisome proliferator-activated receptor (PPAR)-γ [82].

A dose-dependent reduction of HCC incidence was observed in two cohorts of patients: in the first one were diabetics (ORs, 0.32 to 0.53) and in the second one were those infected with HBV (HR, 0.34 to 0.66) and HCV (HR, 0.33 to 0.66) respectively [83,84].

Some data suggest that statins have different effects on chemoprevention, and for this reason, randomized trials are currently underway to determine their role in chemoprevention. In a systematic pair-wise comparison, fluvastatin was shown to be more effective in reducing HCC risk (RR, 0.55) compared to other statins [85], whereas atorvastatin and fluvastatin were associated with more significant anti-fibrotic effects [86].

The high HCC risk in association with type 2 diabetes has been widely discussed above, so anti-diabetic therapies may be rational HCC chemopreventive strategies. Metformin, a biguanide derivate, is an insulin sensitizer drug that inhibits gluconeogenesis and elicits various anti-neoplastic effects.

Metformin inhibits the mammalian target of rapamycin (mTOR) pathway via activation of AMPK and its upstream regulator LKB1 [87] inhibits angiogenesis via suppression of vascular endothelial growth factor (VEGF) and hypoxia inducible factor 1 α (HIF1A) [88], suppresses cell survival-conferring NF-κB signaling by upregulating IκBα [89], and induces apoptosis via p53-independent mechanism [90]. Moreover, metformin suppresses progenitor/stem cell activation, thereby minimizing HCC development in a mouse model of cirrhosis-driven carcinogenesis. Although the chemoprevention effect is observed only when metformin treatment is started before development of cirrhosis [91].

These data are supported by a meta-analysis of 19 studies involving 550,882 diabetic subjects. This suggested that metformin use reduced HCC incidence (OR, 0.52) compared to the control group [92]. In exploratory subgroup analysis, metformin was protective against HCC in patients with HBV/HCV infection (OR, 0.50), cirrhosis (OR, 0.49), and obesity (OR, 0.42). A phase 3 trial (NCT02319200) to evaluate secondary HCC chemopreventive effect of metformin in compensated HCV cirrhosis and insulin resistance was discontinued on the decision of investigator. Another phase 2 trial (NCT02306070) is planned to evaluate change in liver fibrosis by metformin in HCV-infected patients with or without HIV.

### 5.3. Anti-fibrotic Therapies

The rational of use of anti-fibrotic therapies as HCC chemoprevention is supported by halting progression of fibrosis, one of the most independent risk factor of carcinogenesis.

Any chronic liver injury leads to release of inflammation molecules (including TNF, IL6, IL1β, ROS and hedgehog ligands) as a triggers of fibrogenesis by hepatic stellate cell activation [93].

A phase 2 trial (NCT02466516) of ASK1 inhibitor, selonsertib (GS-4997), reduced liver fibrosis (>1 stage) in 43% of NASH patients. Cenicriviroc, a dual inhibitor of fibrosis-promoting CCR2/CCR5 reduced liver fibrosis in a phase 2 trial (CENTAUR) [94], and is now being tested in a follow-up phase 3 trial (AURORA, NCT03028740). A PPARα/δ agonist, elafibranor, stopped fibrosis progression in non-cirrhotic NASH in a phase 2 trial [95], and a follow-up phase 3 trial is ongoing (RESOLVE-IT, NCT02704403). Despite the promising results, the framework for assessing anti-fibrotic therapies for clinically meaningful HCC chemopreventive effects has not yet been established.

## 6. Future perspectives

In this scenario, according to the growing interest of HCC and NAFLD, it is worthy identifying and implementing screening strategies to increase surveillance adherence and new screening tools or devices.

### 6.1. miRNA

There is a growing body of evidence suggesting that circulating microRNAs (miRNAs) play a prominent role as pathogenic factors and diagnostic and prognostic cutting edge biomarkers in NAFLD. miRNAs are small non-protein coding RNA molecules (19–22 nucleotides of length) which regulate cross-talk processes between cells both silencing or enhancing target mRNAs and consequently protein synthesis [96].

miRNAs have been detected in biological liquids (plasma, serum, saliva and urine), are usually associated with proteins or packaged in exosomes [97], and thus are stable and protected from RNase degradation. They might be released both passively during cell death or actively from cells [98]. Among miRNAs, the liver-specific miR-122, miR-34a and miR-16 were recently found increased in serum of NAFLD patients and their expression was associated with liver enzymes, inflammation and fibrosis [99], thus suggesting a potential role as non-invasive biomarkers in the progression from steatosis to NASH [96].

These data were confirmed by Halasz et al., reporting that miR-122 is negatively correlated with liver fibrosis as detected by histology and hepatic stiffness [100]. In addition, miR-122 decrease is associated with fibrotic pathways upregulation by inducing hypoxia-inducible factor 1-α (HIF1α) and mitogen-activated protein kinase 1 (MAPK1) [101].

Furthermore, Guo et al. reported miR-301a-3p, miR-34a-5p and miR-375 as altered liver patterns associated with NAFLD severity in terms of altered lipid and carbohydrate metabolism [102].

Analyzing a panel of 84 miRNAs, Pirola et al. identified that miR-122, miR-192 and miR-375 were dramatically enhanced in NASH compared to simple steatosis [96,103].

As described by Moshiri et al., HCC patients also present with elevated plasmatic levels of miR-106b-3p, miR-101-3p and miR-1246 when compared to healthy control subjects, confirming the utility of these three biomarkers for early detection of HCC in high-risk subjects [104].

Although several miRNA candidates have been identified as differently involved in the progression of liver disease, some observations are inconsistent in certain cases due to patients’ inter-individual variability, environmental factors, and HCC genetic and etiological background. Another contradiction is the evidence of circulating miRNAs with different expression in plasma/serum and in tumor.

At the same time, the pathogenesis of fibrosis and tumorigenesis still need further investigation to identify proper prevention strategies and therapeutic options [69,105].

Recent findings have shown, both in mice and humans, the key role of miR-223 in regulating the progression of steatosis to NASH by a mechanism involving neutrophil maturation and activation. miR-223 avoids evolution to NASH by targeting inflammatory genes such CXCL10 and TAZ, thus exerting anti-infiammatory effects in hepatocytes. Therefore, the overexpression of miR-223 might be considered a potential therapeutic target for the NASH treatment [106].

However, and based on these discoveries, the identification of specific miRNAs associated with the severity of liver disease might be considered an encouraging non-invasive strategy for screening, surveillance and treatment of NAFLD and HCC [107].

### 6.2. lncRNA

HCC, as well as other malignancies, is characterized by a progressive collection of genetic and epigenetic alterations.

Recently, long non-coding RNAs (lncRNAs) have emerged as regulators of the onset and progression of HCC. lncRNAs are ubiquitous transcripts (with a length of 200 bp), characterized by cell-type specificity and tumor-type specificity. A great body of evidence highlights the relationship between lncRNAs and HCC and their role as biomarkers in HCC diagnosis and outcome prediction.

For this purpose, several databases have been created to facilitate research into these newly discovered biomarkers and their interaction with other RNAs in conditioning the initiation, proliferation, apoptosis processes and angiogenesis of cancer [108].

LINC00052, ZEB1-AS1 and LINC01225 have displayed oncogene properties by facilitating the invasiveness and metastasis induction of HCC cells [109,110,111].

HCCL5, a novel cytoplasmic lncRNA, is activated by ZEB1 and promotes cell growth, G1-S transition, HCC cell invasion and metastasis while protecting cancer cells from apoptosis [112].

Some other lncRNAs, such as XIST, HOST2, HOXA-AS2, CCHE1, and AFAP1-AS1, can induce and accelerate cell proliferation while inhibiting apoptosis of HCC cells [108]. This feature might be useful as a therapeutic target of HCC.

LncRNAs may also act in chemo-sensitivity or radio-resistance by blocking the cell cycle, suppressing apoptosis and reinforcing the DNA injury repair [108]. For this reason, they can be used as potential targets for discovering new approaches to chemotherapy and radiotherapy in HCC-affected subjects, as demonstrated by Huang H. et al., who described that LncRNA NR2F1-AS1 regulates HCC oxaliplatin resistance by targeting ABCC1 via miR-363 [113].

Another important role of lncRNAs is exerted in autophagy, the physiological cell pathway to maintaining cell homeostasis. In this context, LncRNA HULC has been identified as an inductor of protective autophagy [108]. In conclusion, these lncRNAs look to be promising tools for HCC diagnosis, prognosis and surveillance, and might be useful as targets for novel therapeutic approaches in HCC.

### 6.3. Exosomes

Recently, a role has been proposed for exosomes in liver diseases, and several studies are ongoing to better understand their potential in new diagnostic and therapeutic approaches.

Exosomes are small vesicles (30–100 nm in size) derived from endosomal membranes, and it was originally thought that they acted as removers of cell debris. Further studies have shown their role in intercellular communication and the modulation of cellular functions. Exosomes are involved in HCC progression by regulating proliferation, angiogenesis and invasion of the tumoral cells. Exosomes may also regulate HCC hypoxia stress and drug resistance. Based on these observations, exosomes are emerging as novel biomarkers of liver diseases [114].

Conigliaro et al. showed that the exposure of endothelial cells to CD90+ exosomes may increase the number and length of vascular structures [115]. On the other hand, due to their biological safety and stability potential, exosomes might be implemented for drug delivery, as well as in vaccination. Nevertheless, further studies on exosomes are needed to figure out their physiological and pathological role in liver diseases and their potential application as novel non-invasive biomarkers in NAFLD and HCC.

### 6.4. Epigenetic

Epigenetic modifications consist in chromosomes alterations without changes in DNA sequence and they have been proposed as a possible molecular explanation for the heterogeneity in individual susceptibility to developing cirrhosis, HCC, and end-stage liver disease [116]. Chromatin remodeling may be a critical step in the progression of simple steatosis to NASH and HCC, through impaired regulation of pro-inflammatory cytokines; overexpression of ATP-dependent chromatin remodeling proteins Brg1 and Brm was observed in hepatocytes cultured with free fatty acids [117]. These proteins are involved in chromatin remodeling through the activation of pro-inflammatory genes and the stabilization of the nuclear factor kappa B (NF-κB) binding. Mice models showed that experimentally depleting Brg1 was associated with the suppression of steatosis, inflammation and fibrosis. DNA methylation represents one of the primary relevant regulatory mechanisms in epigenetic modifications, and it is involved in the pathogenesis of several human neoplasms. Interestingly, using whole-genome DNA hydroxymethylation, a recent study compared AFP-negative HCC to adjacent non-neoplastic tissue, identifying 615 differentially hydroxymethylated regions [118]. The genes associated with these regions exhibited significantly enrichment with respect to functions such as actin binding and vascular morphogenesis, as well as in the MAPK pathway. Particularly, the authors identified key hydroxymethylated genes involved in the regulation of chromatin that could be associated with the occurrence of HCC in the absence of a significant elevation in AFP levels, suggesting their use as potential biomarkers. Although the majority of evidence on epigenetic modifications has been obtained in HBV- and HCV-related liver disease, their evaluation in patients with NAFLD appears to be an attractive tool for improving the personalization and the effectiveness of the surveillance programs and of the future treatments.

## Figures and Tables

**Table 1:** Genetic findings associated with NAFLD-related HCC.

SNP	Number of Patients	Study Design	Cirrhosis	Country	Covariate Adjustment	Reference
PNPLA3 I148M (rs738409, C > G)	100 with HCC-related NAFLD 275 NAFLD controls without HCC	Case-control, retrospective	24.8%	UK and Switzerland	Age, sex, BMI, diabetes, cirrhosis	[29]
PNPLA3 I148M (rs738409, C > G)	471 with NAFLD	Cohort study, propspective	34.4 % (F3–F4)	Italy	Age, BMI, platelet count, albumin, IFG/diabetes, fibrosis F3–F4	[30]
TM6SF2 E167K (rs58542926, C >T)	511 with liver disease (44% alcohol) 228 controls	Case-control, retrospective	100%	Italy	NA	[36]
MBOAT7 (rs641738, C > T)	132 with NAFLD 633 controls	Case-control, retrospective	27.5% (F3–F4)	Italy	Age, sex, obesity, diabetes, fibrosis F3–F4, PNPLA3, TM6SF2	[39]

Abbrevations: SNP, single nucleotide polymorphisms; NAFLD, nonalcoholic fatty liver disease; HCC, hepatocellular carcinoma; IFG, impaired fasting glucose; BMI, body mass index; NA, not assessed.
